# Functional Outcome After Surgery for Popliteal Artery Entrapment Syndrome

**DOI:** 10.1016/j.ejvsvf.2026.01.002

**Published:** 2026-01-14

**Authors:** Lucille Treil, Salomé Kuntz, Nabil Chakfe, Anne Lejay

**Affiliations:** a Vascular Surgery and Renal Transplant Department, University Hospital of Strasbourg, Strasbourg, France; b GEPROMED, Strasbourg, France; c Biomedicine Research Center of Strasbourg, Research Unit 3072 – Mitochondria, Oxidative Stress and Muscular Plasticity, Strasbourg, France

**Keywords:** Functional status, Popliteal artery entrapment syndrome, Vascular surgical procedure

## Abstract

**Introduction:**

Popliteal artery entrapment syndrome (PAES) is a rare but underdiagnosed vascular disorder in young, active individuals, frequently athletes. Although surgery is considered the gold standard, data on functional outcomes, return to sport, and long term quality of life remain scarce. This study aimed to evaluate the functional outcomes of the surgical management of PAES using validated patient reported measures.

**Method:**

Pre- and post-operative functional assessments were performed using the VascuQOL (Quality of Life)-25 questionnaire, complemented by sport specific questions. Imaging (dynamic duplex ultrasound, computed tomography angiography, magnetic resonance imaging when indicated) guided diagnosis was according to the Whelan classification. Primary outcomes were changes in VascuQOL scores; secondary outcomes included arterial patency, return to physical activity, and correlations with PAES type.

**Results:**

Fourteen patients (18 limbs) were analysed (mean age, 30.3 ± 13.6 years; ten men and four women). Most patients presented with exertional claudication; imaging identified predominantly type VI (functional) and type V PAES. VascuQOL-25 scores demonstrated statistically significant improvements in activity, symptoms, pain, and social domains, with overall total score increasing from 4.3 ± 1.2 to 5.2 ± 1.5 (*p* = 0.002). At a mean follow up of 70.4 months, one and five year primary patency rates were 94.4%. Nevertheless, only six of 13 previously active patients returned to pre-symptomatic athletic levels.

**Conclusion:**

Surgical treatment of PAES yields excellent long term patency and significant quality of life gains. However, return to pre-symptomatic athletic performance was limited, highlighting the importance of realistic counselling, tailored rehabilitation, and multidisciplinary management strategies to optimise recovery.

## HIGHLIGHTS

• Surgery for Popliteal Artery Entrapment Syndrome (PAES) provides favourable functional results
• The VascuQOL score significantly improves after PAES surgery.
• Surgery tends to benefit young athletes with non-functional PAES.
• Primary patency of PAES surgery is 94% at five years.

## INTRODUCTION

Popliteal artery entrapment syndrome (PAES) is a rare but clinically significant vascular disorder characterised by the compression of the popliteal artery (PA) by adjacent musculoskeletal structures, most frequently the medial head of the gastrocnemius muscle.^1^ This compression can lead to dynamic arterial stenosis, occlusion, or post-stenotic aneurysmal degeneration with consequent symptoms of lower limb ischaemia. PAES typically affects young and physically active individuals, particularly male athletes, and is considered an important, but frequently overlooked, cause of exertional leg pain in this population.^2^^,^^3^

The prevalence of PAES is estimated to be between 0.16% and 3.5% in the general population,^1^^,^^4^ but its true incidence is difficult to ascertain because of its under recognition and frequently delayed diagnosis. The physiopathology involves either congenital anatomic abnormalities or functional muscular hypertrophy that leads to dynamic extrinsic compression of the PA during repetitive movement, especially knee flexion. The Whelan classification system distinguishes several types of anatomic PAES based on the nature and location of the vascular entrapment. In addition to these anatomic forms (types I – V), a functional variant, referred to as type VI, is increasingly recognised, wherein no structural anomaly is present but muscular hypertrophy or dynamic malposition causes intermittent compression.^1^^,^^5^^,^^6^

Once diagnosed, surgical decompression remains the gold standard for symptomatic PAES. The surgical approach is tailored to the type of entrapment and the degree of vascular injury. Options include PA freeing, release of the compressing musculotendinous structures, myotomy, resection of aberrant bands, and, when necessary, arterial reconstruction using autologous vein grafts.^7^^,^^8^ Several studies have demonstrated favourable technical outcomes, including high primary patency rates and low peri-operative complication rates. However, the literature on functional recovery, return to sport, and long term quality of life after surgery remains sparse.^9^

In physically active populations, particularly athletes, restoration of functional capacity and return to pre-symptomatic levels of activity are critical measures of successful treatment. Although anatomic success is often assumed to correlate with functional recovery, there are limited data specifically quantifying this relationship using validated outcome measures. Understanding how surgical intervention impacts long term function is essential for informed patient counselling and post-operative rehabilitation planning.

In this context, this study aimed to evaluate the functional outcomes of the surgical management for PAES. The records of a cohort of patients who underwent surgery for PAES were reviewed retrospectively, and pre- and post-operative functional score data were collected and compared. The goal was to evaluate the results of surgery for PAES.

## STUDY DESIGN

This retrospective cohort study included all patients who underwent PAES surgical treatment between January 2010 and December 2023 and answered the functional outcome questionnaire before and after surgery. Patients were prospectively identified, and a review of all files was performed.

## METHOD

### Pre-operative parameters and popliteal artery entrapment syndrome classification

Pre-operative evaluation of patients included history of reported symptoms, limb affected, duration of symptoms, impacts on the day to day life, professional occupation, and pre-symptomatic level of physical activity.

Imaging studies were standardised with duplex ultrasound (DUS) before and after dynamic manoeuvres, looking at PA compression, downstream blood flow impairment, and potential intraluminal PA lesions. Computed tomography angiography (CTA) was systematically performed before and after dynamic manoeuvres, looking for PA compression, anatomic anomalies of the popliteal fossa, endothelial lesions, or PA occlusion. In some cases, magnetic resonance imaging was performed to look more closely at the musculotendinous insertions in the popliteal fossa.

Dynamic manoeuvres consisted of ankle dorsi- and plantar flexion performed while standing until symptoms began or for a maximum of five minutes. With CTA, the manoeuvres can be maintained during image acquisition by placing a block beneath the feet of the patient on the CTA bed and asking them to maintain either ankle dorsiflexion or plantar flexion.

The anatomic imaging findings were used to classify the anatomic type of PAES for every limb according to the modified Whelan classification. The pre-operative PAES classification was compared with per-operative findings and confirmed or modified accordingly.

### Per- and post-operative parameters and follow up

Per-operative findings were documented, including the anatomic structures responsible for arterial compression, the extent of arterial involvement, and any need for arterial reconstruction. Surgical techniques varied according to the Whelan type that included PA freeing, myotomy, fibrous band release, or arterial bypass with autologous vein grafting in cases with arterial occlusion.

Surgical procedures were recorded, and post-operative complications were recorded during the first 30 days. Post-operative rehabilitation was supervised by a physiotherapist. No specific post-operative rehabilitation protocol was used. Post-operative follow up included systematic clinical assessment at six weeks and imaging studies to evaluate arterial patency. DUS with dynamic manoeuvres was routinely performed at one and 12 months after surgery to assess vessel integrity and exclude re-stenosis or thrombosis. Follow up after one year was left to the referring physician's discretion.

### Functional assessment

Functional assessment was performed using a multiple choice questionnaire including 25 questions evaluating quality of life (QOL): the VascuQOL-25 questionnaire. For each question, seven answers are offered and every answer is assigned a score from one to seven, one being representative of the most symptomatic patient and seven being representative of the least symptomatic.^10^ The final result of the questionnaire is presented with six scores corresponding to different aspects of the day to day life of the patient: activity score, symptoms score, pain score, emotional score, social score, and total score. These scores are the mean of the individual scores for the questions targeted towards the different aspects of the day to day life of the patient.

The activity score included questions about ability to exercise, evolution of walking distance, ability to walk, ability to climb stairs, ability to do household work, perceived range of activities, and ability to go shopping.

The emotional score included questions about worry about injuries, concerns about being housebound, concerns about having poor circulation in the legs, frustration about problems caused by poor circulation in the legs, feelings of guilt about relying on friends or relatives, worries about being in danger of losing a part of a leg or foot and being depressed about poor circulation in the legs.

The pain score included questions about pain while walking, pain at night, pain at rest, and amount of discomfort.

The social score included questions about restriction in time spent with friends or relatives and ability to participate in social activities.

The symptoms score included questions about presence of cold feet, tired or weak legs, pins, needles or numbness, ulcers, and sores.

Additional questions were added to the questionnaire, including what sport activity was practised before surgery, whether return to activity was possible after surgery, and whether return to pre-operative level of physical activity was possible. Patients were asked to fill out the VascuQOL-25 once for their pre-operative status and once for their post-operative status at three months from surgery.

### Outcomes

The primary outcome was functional score change between the pre- and post-operative periods. Secondary outcomes included one year arterial patency, sport practice ability, and correlation between type of PAES (functional or other) and functional score improvement.

### Statistical analysis

Data were analysed using JASP (version 0.95.1) (University of Amsterdam, Amsterdam, The Netherlands) (computer software). Continuous variables were expressed as mean ± standard deviation or median with interquartile range, depending on distribution. Categorical variables were presented as percentages. Paired Wilcoxon signed rank tests and repeated measures analysis of variance were used to compare pre-operative and post-operative functional scores. Spearman correlation coefficients evaluated the relationship between arterial patency and functional improvement. A p value < .050 was considered statistically significant.

## RESULTS

### Patients

Between 1 January 2010 and 31 December 2024, 18 patients were identified (24 limbs), but only 14 patients (18 limbs) with complete functional assessment were included. There were ten men and four women. Four patients were treated bilaterally, making 18 limbs treated. The mean age at the time of surgery was 30.3 ± 13.6 years.

### Pre-operative assessment

The clinical presentation during the first outpatient clinic visit was claudication while walking for nine patients (ten limbs) and while practising physical activity for five patients (seven limbs). One limb was asymptomatic at the time of surgery in a professional athlete with contralateral impairment.

The mean duration of symptoms before first surgical consultation was 14.4 ± 9.2 months. Thirteen patients enjoyed physical activity before the appearance of symptoms, one being a professional athlete, one having a high intensity job (professional army career), and three being in a pre-professional training programme. Regarding the type of physical activity, five patients played football, two basketball, one snowboarding, one cycling, and one was a runner. Complete description of the population is shown in Table 1.

**Table 1:** Population description.

Patient	Age – y	Sex	Side	Presentation	Duration – mo	Sport	Pro Athlete	d-DUS	d-CTA	MRI	PA Lesion	Type
1	34	M	R	Claudication	9	Snowboard	Y	+	+	N	<15 mm post-stenotic ecstasy	V
	34	M	L	Asymptomatic	0	Snowboard	Y	+	+	N	–	V
2	22	M	R	Claudication	18	Football	N	+ on DUS	+	+	–	III
3	56	M	L	Claudication	9	Football	N	N	+	N	–	VI
4	22	F	L	Claudication	36	Running	Y	+	+	+	–	VI
5	34	M	R	Claudication	18	Running	N	N	+	N	–	V
6	23	F	R	Claudication	24	Basketball	N	+	+	N	–	VI
	23	F	L	Claudication	24	Basketball	N	+	+	N	–	VI
7	28	M	L	Claudication	9	Football	N	+	+	N	–	VI
8	55	M	R	Claudication	14	–	N	+	+	N	–	VI
9	19	F	L	Sports pain	22	Running	N	+	+	N	–	VI
	19	F	R	Sports pain	23	Running	N	+	+	N	–	VI
10	17	M	L	Sports pain	3	Football	N	+	+	N	–	VI
	17	M	R	Sports pain	3	Football	N	+	+	N	–	VI
11	20	M	R	Sports pain	18	Football	Y	+	+	N	–	V
12	17	M	L	Sports pain	8	Basketball	Y	+	+	+	–	V
13	29	M	L	Sports pain	10	Running	Y	+	+	+	–	II
14	48	F	R	Claudication	12	Biking	N	+	+	N	Segmental occlusion of PA (<2 cm)	V

Pro = professional; d-DUS = dynamic duplex ultrasound; d-CTA = dynamic computed tomography angiography; MRI = magnetic resonance imaging; PA = popliteal artery; M = male; R = right; Y = yes; + = performed and positive; N = no or not performed; L = left; DUS = duplex ultrasound; F = female.

CTA demonstrated extrinsic compression of PA in all limbs (one at rest and 13 after dynamic manoeuvres). One limb had an intimal PA lesion, and one presented with segmental PA occlusion.

Based on the imaging results, there were ten type VI limbs, six type V, one type III, and one was type II according to the modified Whelan classification.

Complete description of pre-operative workup is available in Table 1.

### Per-operative data, post-operative complications, and follow up

All procedures were performed in a prone position with posterior access to the popliteal fossa. All PAs were liberated from constraint by ligating the afferent collateral until the sural arteries and by resecting all fibrous PA restraints. In 11 limbs, patients benefited from concomitant popliteal vein collateral and fibrous restraint liberation. Muscle resection consisted of resection of an abnormal insertion of the medial gastrocnemius muscle in seven limbs, resection of an accessory slip of the medial gastrocnemius muscle in one limb, and partial resection of the medial head of the gastrocnemius muscle (between 25 and 50 mL) in ten limbs. Resection of the tibial insertion of the soleus muscle and opening of the leg compartments was performed in three limbs. In one case, a PA endarterectomy with venous patch closure was performed. The mean intervention duration was 94.2 ± 42 minutes.

Systemic heparinisation and completion angiography were performed in the case having vascular reconstruction.

The mean hospital stay was 3.3 ± 1.0 days. Thirty day morbidity consisted of an isolated deep vein thrombosis in one patient and post-operative neuropathic pain in another.

### Evolution of functional assessment

Comparison of the pre- and post-operative VascuQOL-25 functional assessment found significant score improvements for individual questions (Table 2).

**Table 2:** Median score for every Vascular Quality of Life Questionnaire question before and after surgery with associated results of paired Wilcoxon test.

Question	Pre-operative median score (SD)	Post-operative median score (SD)	*z*	*p* value
VascuQOL Q1	2.0 (4.25)	4.5 (2.75)	−2.376	0.009
VascuQOL Q2	4.0 (4.5)	6.0 (3.5)	−1.067	0.15
VascuQOL Q3	4.0 (2.5)	6.5 (3.0)	−2.934	0.002
VascuQOL Q4	2.0 (1.0)	5.0 (4.0)	−3.180	<0.001
VascuQOL Q5	3.5 (4.0)	6.0 (2.0)	−2.401	0.009
VascuQOL Q6	6.5 (3.0)	7.0 (0.75)	−2.366	0.011
VascuQOL Q7	4.5 (5.0)	5.0 (3.0)	−1.245	0.11
VascuQOL Q8	3.0 (4.0)	5.0 (2.75)	−1.867	0.033
VascuQOL Q9	1.0 (0)	2.0 (2.75)	−2.785	0.003
VascuQOL Q10	3.0 (3.75)	6.0 (3.0)	−2.934	0.002
VascuQOL Q11	6.5 (3.0)	7.0 (0.75)	−1.680	0.049
VascuQOL Q12	2.5 (2.0)	6.0 (2.75)	−2.574	0.005
VascuQOL Q13	4.0 (5.0)	6.0 (2.75)	−2.201	0.018
VascuQOL Q14	4.0 (3.0)	7.0 (3.0)	−2.666	0.004
VascuQOL Q15	6.5 (2.0)	7.0 (0)	−2.366	0.009
VascuQOL Q16	7.0 (2.0)	7.0 (0)	−2.023	0.028
VascuQOL Q17	7.0 (0)	7.0 (0)	1.604	0.97
VascuQOL Q18	3.0 (1.0)	6.0 (1.0)	−3.621	<0.001
VascuQOL Q19	3.0 (2.0)	4.5 (2.75)	−2.746	0.003
VascuQOL Q20	3.0 (2.75)	5.5 (3.0)	−2.934	0.002
VascuQOL Q21	7.0 (1.0)	7.0 (1.75)	0.178	0.59
VascuQOL Q22	6.5 (3.0)	7.0 (1.75)	−0.612	0.29
VascuQOL Q23	6.0 (2.0)	7.0 (3.0)	−0.355	0.38
VascuQOL Q24	3.5 (4.5)	5.0 (2.0)	−2.291	0.011
VascuQOL Q25	3.0 (6.0)	6.0 (3.0)	−1.883	0.032

SD = standard deviation; Q = question.

For the pre-operative functional assessment, the mean activity score was 3.8 ± 1.2, mean symptom score was 4.5 ± 1.6, mean pain score was 3.9 ± 1.8, mean emotional score was 4.5 ± 1.2, mean social score was 5.6 ± 1.5, and mean total score was 4.3 ± 1.2.

For the post-operative functional assessment the mean activity score was 5.0 ± 1.4, mean symptom score was 5.3 ± 1.9, mean pain score was 5.0 ± 1.9, mean emotional score was 5.3 ± 1.6), mean social score was 6.4 ± 1.0, and mean total score was 5.2 ± 1.5.

**Figure 1:**
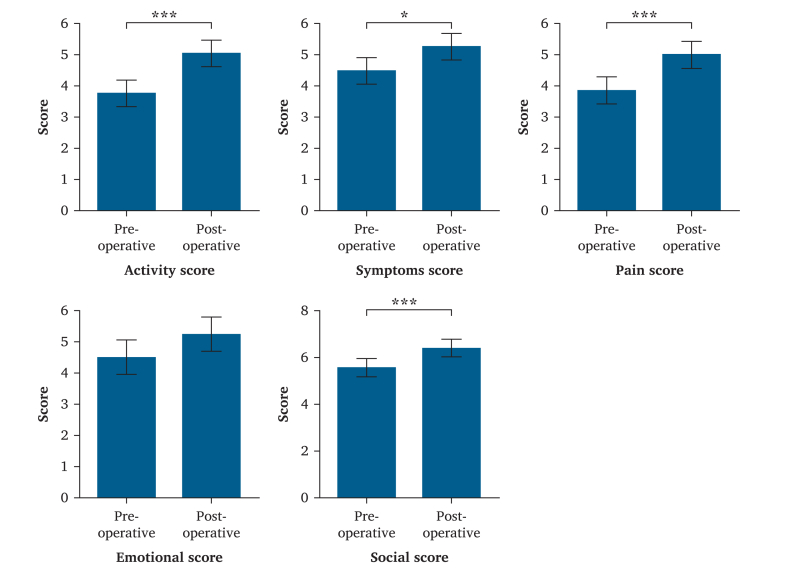
Bar graphs of analysis of variance results regarding the Vascular Quality of Life Questionnaire score difference between pre- and post-surgical procedure. ∗ <.05. ∗∗∗ <.001.

The paired sample analysis of variance found significant improvement in the activity score (*F* = 19.80; *p* ≤ 0.001), symptom score (*F* = 7.64, *p* = 0.013), pain score (*F* = 15.03; *p* = 0.001), and social score (*F* = 10.63; *p* = 0.005). There was no significant difference in the emotional score (F=4.05; p=.065) as shown in Fig. 1.

**Figure 2:**
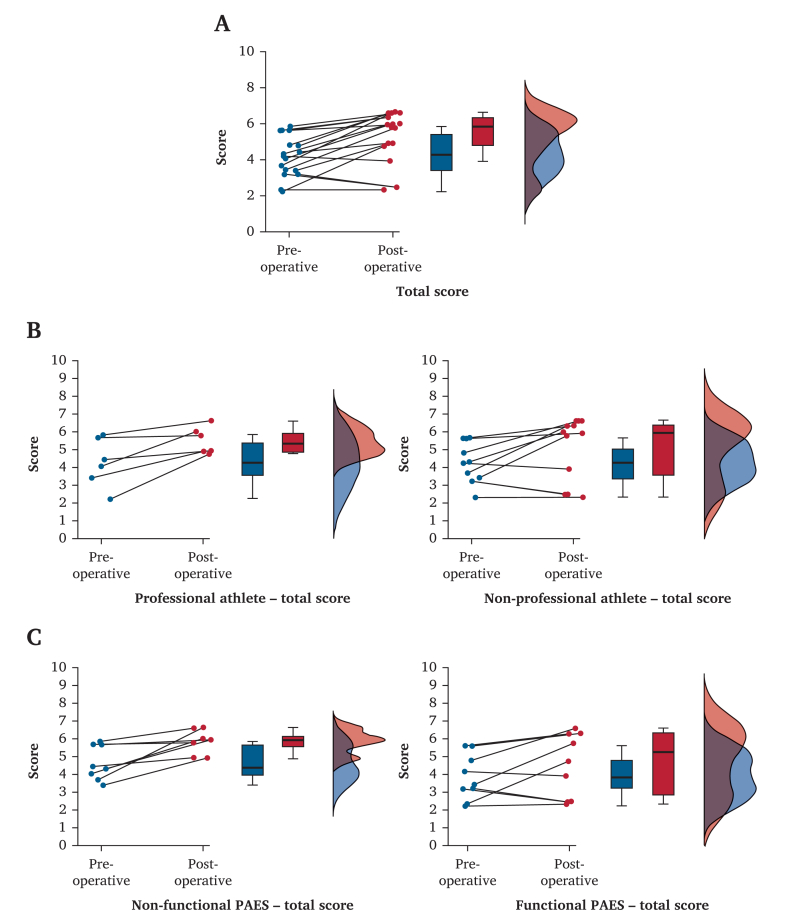
(A) Raincloud plot of the total score distribution. From left to right, distribution of the pair of total score for every patient, box plot of the pre- and post-operative total score for the population (median pre-operative total score 4.260, median post-operative total score 5.840), and distribution of the pre- and post-operative total score. (B) Raincloud plots of the total score distribution in professional and non-professional athletes. From left to right for every plot, distribution of the pair of total score for every patient in the group, box plot of the pre- and post-operative total score for the population (median pre-operative total score for professional athlete 4.240, median post-operative total score for professional athlete 5.340, median pre-operative total score for non-professional athlete 4.260, median post-operative total score for non-professional athlete 5.960), and distribution of the pre- and post-operative total score. (C) Raincloud plots of the total score distribution in patients with functional and non-functional popliteal artery entrapment syndrome (PAES). From left to right for every plot, distribution of the pair of total score for every patient, box plot of the pre- and post-operative total score for the population (median pre-operative total score for non-functional PAES 4.380, median post-operative total score for non-functional PAES 5.940, median pre-operative total score for functional PAES 3.820, median post-operative total score for functional PAES 5.260), and distribution of the pre- and post-operative total score.

Total score results demonstrated a significant improvement (*F* = 14.17; *p* = 0.002). As shown in Fig. 2A, most patients demonstrated improvement in total score, but some did not, and some worsened.

### Correlation between functional score and physical activity

Three patients presented with recurrence of symptoms without confirmed re-stenosis or PA occlusion on dynamic DUS or dynamic CTA. Of the 13 patients who enjoyed physical activity before beginning symptoms, 12 could return to their favoured physical activity, but only six recovered their pre-operative sports level.

As shown in Fig. 2B, patients with a professional level of physical activity before symptoms tended to have a better post-operative total score (*F* = 0.330; *p* = 0.57) with distribution of the total score towards the higher scores. On the other hand, patients who were not professional athletes tended to have little to no improvement in the total score.

As shown in Fig. 2C, patients with type VI PAES, also known as functional PAES, tended to have less improvement in their post-operative total score (*F* = 0.526; *p* = 0.48).

There was no significant correlation between the total score difference and pre-symptom athlete status (ρ = 0.182; *p* = 0.49) or between pre-symptoms athlete status and return to previous level of physical activity (ρ = 0.358; *p* = 0.17), but there was a significant negative correlation between functional PAES and return to previous level of physical activity (ρ = −0.630; *p* = 0.004).

### Follow up

Mean follow up was 70.4 ± 50.5 months. There were no patients lost to follow up.

**Figure 3:**
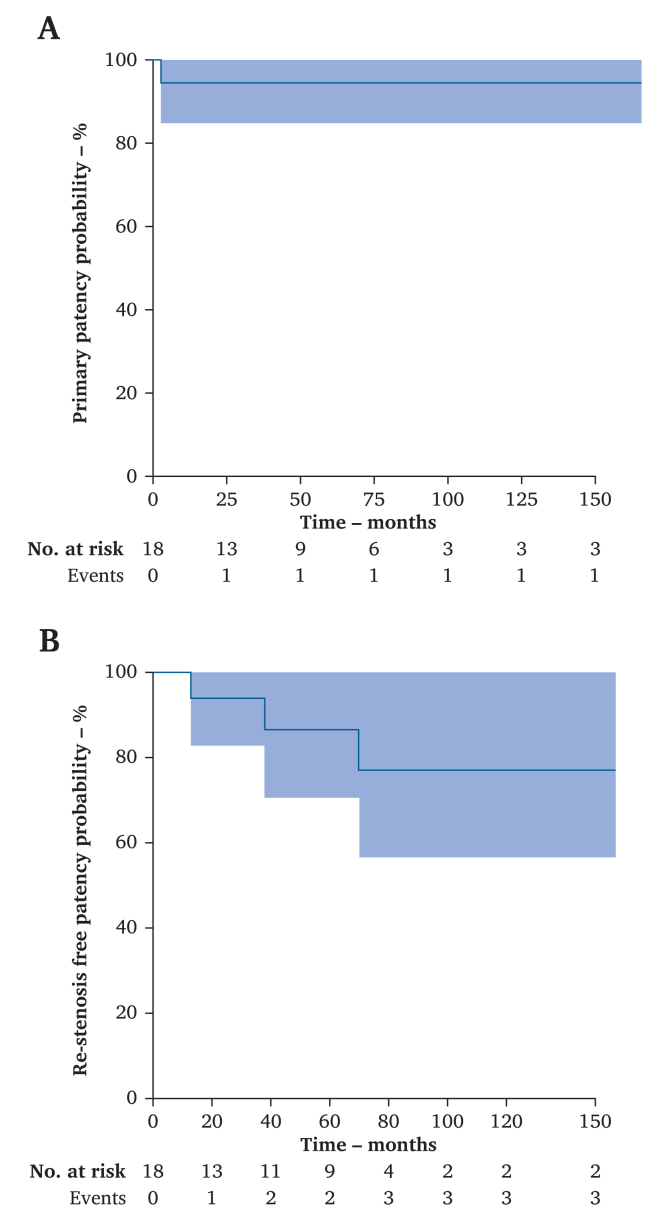
Cumulative Kaplan–Meier estimate of (A) primary patency and (B) re-stenosis free patency.

The one year primary patency was 94.4% with one case of PA occlusion successfully treated with intra-arterial thrombolysis and balloon angioplasty. Five year primary patency was 94.4%, as shown in Fig. 3A.

One year and five year freedom from re-stenosis were 100% and 88.9%, respectively (Fig. 3B).

The three limbs that presented with re-stenosis were all diagnosed by DUS with dynamic manoeuvres, but only one was confirmed with dynamic CTA. In two limbs, which were both type V PAES initially, re-stenosis was associated with the recurrence of symptoms. One limb was treated with a saphenous vein femoropopliteal bypass because of the extent of the intimal damage of the PA, and the other was treated by further freeing of the PA by additional muscle resection. The third limb, which was type VI initially, was asymptomatic and managed with walking rehabilitation and physical therapy.

## DISCUSSION

The main findings of this study are that there is a significant improvement in the functional assessment score after surgery for PAES.

For functional assessment, the VascuQOL-25 was used to get a more complete picture of the impact of PAES on the day to day life of patients. Cabot *et al.*^11^ used the Tergler scale, which is consistent with their population of high level athletes with functional PAES only. In this study, all types of PAES were included in order to assess whether the functional improvement expected from surgery was consistent across all patients with PAES. Here, significant improvements were demonstrated across most domains, pain, symptoms, activity, and emotional impact, thus confirming that surgical anatomic correction does translate into quality of life gains.

However, although many patients were able to resume physical activity, fewer returned to their pre-symptomatic performance levels. This dichotomy is seen in other cohorts focused on functional PAES, but to a lesser degree. One explanation for these results might lie in the inclusion of all PAES types in this cohort compared with only functional PAES in the study from Lavingia *et al.*^12^

It is also important to note that symptoms returned without significant stenosis in three patients, which is consistent with the literature for PAES in the general population outside of targeted cohorts of athletes. This might be explained by the underdiagnosis of chronic exertional compartment syndrome as suggested by Fitzgibbon *et al.*^13^

These findings highlight the importance of setting realistic expectations for athletic individuals and of including sport specific rehabilitation and potentially longer recovery periods in their care pathway. The number of patients presenting with claudication on walking compared with those who presented with pain on exercise, also suggests that the level of pre-procedural impairment was advanced and that recovery to full performance capacity would be expected to be prolonged and might require specific rehabilitation. In this cohort, no specific patient tailored rehabilitation was performed. However, it seems mandatory to create a specific network of rehabilitation professionals for these patients. In fact, the care pathway for patients with PAES needs to be multidisciplinary with the inclusion of sports medicine, physiotherapy, and, in the case of professional athletes, their training professional. This care pathway needs to become multidisciplinary before surgery so the post-operative rehabilitation is anticipated and tailored to the needs of each patient.

Improvement of the post-operative total score seems greater in the professional athlete group than in the non-professional athlete group. When compared with studies on functional PAES, the rate of return to the previous level of activity is low. This suggests that factors beyond arterial patency such as muscular adaptation, reperfusion injury, conditioning, psychological readiness, and perhaps residual damage, play significant roles. To further this point, the high rate of professional athletes returning to their previous level of physical activity in this cohort compared with the non-professional athletes seems to indicate that something in their peri-operative care allows for this better recuperation.

Surprisingly, when looking at the functional PAES in this cohort, they tended to present less improvement in their functional score than the non-functional PAES, which has not been shown in other cohorts, including all types of PAES.^14^ However, systematic testing for associated chronic exertional compartment syndrome was not performed in the centre, but this is a typical differential diagnosis for PAES, and this might explain the more nuanced improvement in the functional PAES population. For these patients, when comparing functional outcomes of surgical management with those of non-surgical management, it was found that outcomes in patients operated on for functional PAES with asymptomatic limbs or mild symptoms were comparable to outcomes when the same patients were managed non-surgically.^15^ In the cohort from Ahn *et al.*,^16^ patients with mild to no symptoms remained stable with non-surgical management in most cases. This raises the importance of a multidisciplinary approach to this pathology with surgical evaluation and physical and rehabilitation medicine evaluation before considering surgery.

The one and five year primary patency rates observed in this series (94.4% in both cases) are consistent with published PAES cohorts. Previous cohorts have reported midterm patency rates in the range of 80–95%, depending on whether arterial reconstruction was involved and on the presence of advanced arterial damage at the time of surgery.^17^, ^18^, ^19^ Moreover, when compared with historical cohorts,^17^^,^^18^ the population appears younger, with less severe PA lesions. This observation is consistent with the recent literature^19^ and with the earlier diagnosis in patients with leg pain while practising physical activity. Thirty day morbidity remained low with only minor complications, although the neurological complication presented by one patient might have impacted their functional assessment questionnaire. This raises the importance of looking further than primary patency to evaluate the surgical outcome in PAES.

The study has several limitations. Firstly, it remains a single centre retrospective study. Secondly, the population is small, and this does not allow for the drawing of a valid conclusion between functional score improvement and professional athlete status. Lastly, the post-operative functional assessment was completed within three months. It might be assumed that the functional results would have been different if the post-procedural questionnaire had been completed at a later stage, especially regarding return to previous physical activity.

### Conclusion

This study suggests that surgical management of PAES can achieve excellent long term vascular patency and meaningful improvements in quality of life and function. However, the limited rate of return to pre-symptomatic athletic performance underscores the need for realistic pre-operative counselling, sport tailored rehabilitation, and possibly longer recovery timelines for high performance athletes.

## Funding

This research did not receive any specific grant from funding agencies in the public, commercial, or not for profit sectors.

## CONFLICT OF INTEREST

No conflicts of interest to declare.
